# Mucociliary Clearance in Mice Measured by Tracking Trans-tracheal Fluorescence of Nasally Aerosolized Beads

**DOI:** 10.1038/s41598-018-33053-2

**Published:** 2018-10-03

**Authors:** Troy D. Rogers, Lawrence E. Ostrowski, Alessandra Livraghi-Butrico, Brian Button, Barbara R. Grubb

**Affiliations:** Marsico Lung Institute, University of North Carolina School of Medicine, Chapel Hill, NC 27599 USA

## Abstract

Mucociliary clearance (MCC) is the first line of defense in clearing airways. In genetically engineered mice, each component of this system (ciliary beat, mucus, airway surface hydration) can be studied separately to determine its contribution to MCC. Because MCC is difficult to measure in mice, MCC measurements are often omitted from these studies. We report a simple method to measure MCC in mice involving nasal inhalation of aerosolized fluorescent beads and trans-tracheal bead tracking. This method has a number of advantages over existing methods: (1) a small volume of liquid is deposited thus minimally disturbing the airway surface; (2) bead behavior on airways can be visualized; (3) useful for adult or neonatal mice; (4) the equipment is relatively inexpensive and easily obtainable. The type of anesthetic had no significant effect on the rate of MCC, but overloading the airways with beads significantly decreased MCC. In addition, the rate of bead transport was not different in alive (3.11 mm/min) vs recently euthanized mice (3.10 mm/min). A 5-min aerosolization of beads in a solution containing UTP significantly increased the rate of MCC, demonstrating that our method would be of value in testing the role of various pharmacological agents on MCC.

## Introduction

Airway clearance of inhaled particulate matter and secreted mucus by beating cilia that line the airways is the first line of defense to maintain clear airways and prevent pulmonary bacterial and viral infections. Effective mucociliary clearance (MCC) requires the coordinated beating of cilia, the correct composition and quantity of mucus, and optimal hydration of the airways^1,2^, yet the interaction between these components to maintain clear airways is still not completely understood. Since the development of genetically engineered mice in which one or more of the components required for effective MCC is either knocked out or in some way altered, there has been much interest in measurement of MCC in mice. These mice have given and will continue to give us valuable information on how the MCC system is integrated and ways to enhance MCC to prevent pulmonary diseases. However, when a component of the clearance system has been altered, it is paramount that mucociliary clearance is quantified to determine the overall effect on MCC and significance of each component. In many murine studies in which various components of MCC have been altered, no measurement of MCC was provided^3–5^. This is likely due to the inherent difficulty of measuring MCC in mice.

Because of their small body size, traditional techniques that are used to measure MCC in larger species are often not readily adaptable to mice. Many of the studies of murine MCC are performed on excised tracheas (e.g.,^6,7^), which can be problematic as hydration, tissue geometry, innervation and mucus production by distal airways have been markedly changed. Studies in which the trachea is left *in situ* but opened longitudinally suffer from the difficulty in keeping the tracheal lumen at the proper temperature and humidity. Other investigations have studied tracheal MCC *in vivo,* but often large volumes of liquid (up to 50 µl) are deposited on the airways^8,9^, thus obviously altering the depth and concentration of the airway surface liquid, which has been shown to be important in maintaining normal MCC^10,11^. Other *in vivo* studies add a minimal amount of liquid to the trachea but require the presence of a microdialysis probe, which necessitates opening the trachea^12,13^. A number of MCC studies, which have provided much useful data, deposit fluorescent microspheres in the mainstem bronchi with only a minimal amount of liquid (200 nl). However, these studies again require a small incision to be made in the upper trachea and the results do not yield a quantitative rate of MCC^14,15^. Other studies employing the synchrotron for MCC measurement are less invasive but the mice require intubation, the mice are exposed to lethal doses of radiation^16^, and in addition synchrotron studies are available to only a few investigators due to the limited worldwide availability of this expensive equipment.

Here we report an *in vivo* method to measure MCC in adult or neonatal mice that does not require opening the trachea to introduce beads into the airways. Specifically, 200 nm fluorescent beads were aerosolized, the mouse inhaled the beads through the nose, and beads were deposited in the nose and the lower airways. Then, the fluorescent beads were tracked through the closed trachea as they traversed rostrally via MCC. The method is relatively inexpensive using easily fabricated or adapted equipment, and the behavior of the beads and beads embedded in mucus can be observed in real time (similar to that in the synchrotron study), which provides additional information, such as whether the beads traverse in a straight or circuitous pathway.

## Material and Methods

All mouse studies were approved by the University of North Carolina Institutional Animal Care and Use Committee. All mouse studies were performed in accordance with the guidelines and regulations governing these animals. Male mice, C57Bl/6 N (Taconic, NY or a limited number of males and females raised at UNC) 8–10 weeks old were used for our studies. A small group of 10-day-old neonatal C57Bl/6 N mice raised at UNC were also studied. All mice were provided food and water ad libitum until they were studied.

We have developed a murine nose-only aerosol delivery system to aerosolize ultra-small volumes (10 µl/min or less) by modifying a commercial ultrasonic aerosol generator (Aeroneb). We reduced the duty cycle of the nebulizer activation and increased the impedance of the control signal to the nebulizer head to reduce the intensity of the piezo “vibrations” controlling nebulizer output. The nebulizer can be used unmodified, but larger volumes will be delivered. Aerosol generators suitable for mice are commercially available (see Supplementary Table S1). The aerosol is delivered via a small conical tip (I.D 4 mm, cut from a 10 ml plastic pipette) placed directly over the nose of an anesthetized mouse. In this study we aerosolized 200 nm beads (ThermoFisher, In Vitrogen, Carboxylate-modified, yellow-green fluorescent (505/515, 2% solids)) for 5 or 15 min. Before use, the beads were washed two times in distilled water (DI) water and then reconstituted at 1×, i.e., 2% solids, 1/4×, i.e., 0.5% solid or 1/3 × 0.67% solids as noted, in an isotonic (305 mOsm) NaCl solution for delivery. A small group of mice was exposed to 7% NaCl (containing 1x beads). We have previously measured the mean particle (droplet) size delivered by the modified system and found it to be ~4 µm (unpublished). Based on studies using the 200 nm beads aerosolized via the nose, knowing the concentration of beads in the aerosol generator reservoir, and the quantity of beads in the lungs, we have estimated that with our system, a 5-min aerosol period delivers ~0.095 µl H_2_O to the lungs/trachea (unpublished) (overall generator output was 9.7 ± 0.44 µl/min (N = 19)). In addition to the charged carboxylate-modified beads, we also studied the transport of pegylated, 100 nm fluorescent beads, a generous gift from Justin Hanes^17^.

The mouse was placed in a Plexiglas chamber (15 cm × 15 cm × 23 cm; Supplementary Fig. S1) into which isoflurane was delivered via a calibrated vaporizer (Forane, Ohio Medical Products, Madison, WI) (flow rate ~3 l/min and ~66% humidity achieved by bubbling the isoflurane through a water column). Most experiments were conducted on mice anesthetized with isoflurane inhalation (see below) but for some studies, Avertin (2.5% tribromoethanol, 100 µl/10 g body mass) injected I.P. was used as an anesthetic. For most experiments, the mouse was anesthetized for approximately 10 min before bead aerosolization was started. The aerosol delivery apparatus was placed inside the isoflurane chamber and the aerosol was delivered through a small conical tip placed directly over the nose of the anesthetized mouse. During aerosolization, a rectal probe was used to monitor the mouse’s body temperature which was kept at 37 °C via a heat lamp connected to a Physitemp temperature controller (Physitemp Instruments, Clifton N. J).

In most studies, the anesthetized mouse was euthanized by severing the aorta immediately post-aerosolization just prior to MCC measurement. (A small group of mice was studied anesthetized, i.e., without exsanguination.) To prepare a mouse for image acquisition, the muscle and connective tissue covering the trachea (~1 cm in length) was retracted to expose the trachea. Usually almost no bleeding occurred, but any small oozing was stopped by blotting, as it could obscure visualization of MCC. A piece of plastic wrap covered in mineral oil was placed on the exposed trachea to prevent water loss. While the body temperature of the mouse was maintained at 37 °C during imaging, the covered, exposed trachea often dropped below body temperature during the image acquisition period (especially in experiments measuring MCC on mice post-euthanasia). Thus a small temperature probe (Eaton, Eutech Instruments, Singapore) was placed alongside the trachea, and the temperature was monitored and adjusted with a heat lamp to keep the *in situ* tracheal preparation at 37 °C. For experiments in which the MCC was measured on a living, anesthetized mouse, the exposed trachea was directly under the microscope and isoflurane was continuously administered by having the nose of the animal inserted into a rubber dam in the anesthesia/aerosolization chamber. Image acquisition was initiated immediately before the aerosolization was begun.

For bead visualization, the preparation was then placed under a dissecting microscope outfitted with a 520 nM long-pass filter (Edmund Scientific) and illuminated with a high-power 470 nm LED (Luxeon). Images were obtained with a CCD camera (MTI, Michigan City, IN) interfaced to a DVD recorder. A slide micrometer was placed on the stage of the dissecting scope to calibrate distance measurements. Once the video was recorded, MCC was determined by measuring the time for groups of fluorescent particles (rafts) to traverse a calibrated distance on the screen monitor (corresponding to 0.5–1 mm *in vivo*). Rafts traversing the calibrated field were manually measured. When a large number of rafts traversed the field simultaneously, not all of them could be measured during a single pass. Thus, the video was re-played as needed to measure the rate of transport of the rafts missed the first time.

*Statistics* All data are shown as means +/− SE. A student’s *t* test was used to compare means between two groups. Chi Square was used to test expected vs observed frequencies. Pearson Product moment correlation was used to determine a correlation between two variables. P ≤ 0.05 was considered statistically significant.

## Results

### Effect of length of aerosolization period

**Figure 1:**
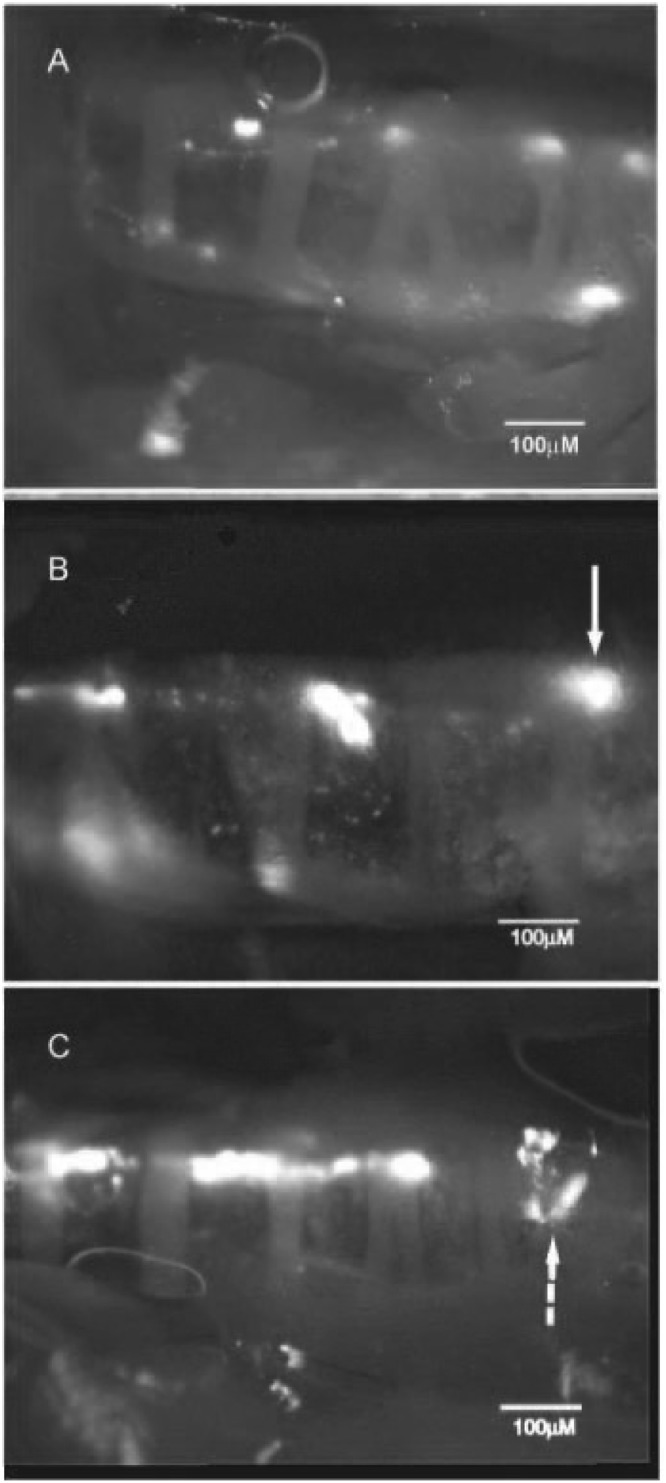
(**A**) Still image captured from video showing rafts (mobile groups of fluorescent beads) transported by MCC toward the epiglottis (beads were moving from left to right). Faintly visible are tracheal rings. Some rafts are out of focus as they are on a different plane of the tracheal wall. All visible fluorescent groups in this preparation were mobile. Some reflection artifacts outside of the trachea are visible. (**B**) In this preparation only one group of beads was mobile (arrow) at the time the image was captured. All other groups of fluorescent beads were immotile, including the very small groups of beads. (**C**) In some mice, the rafts coalesced to form continuous streams, as can be seen in this image. All groups of beads in this image were motile. There are a number of fluorescent areas in each image that are artifacts due to reflections, such as the group of fluorescent reflections on the right side of (**C**) (dashed arrow). Each image is from a different mouse.

**Figure 2:**
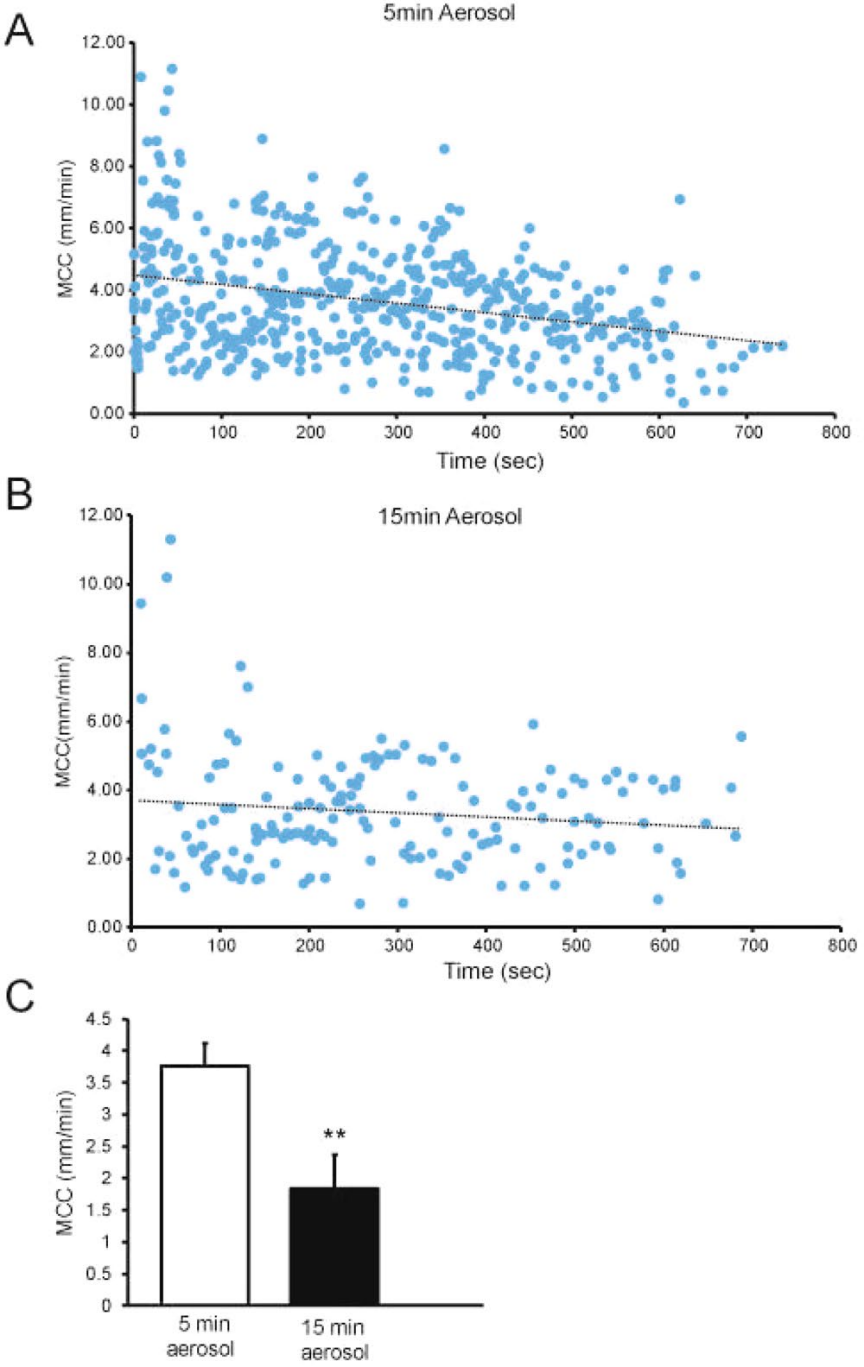
(**A**) Mucociliary clearance measured over ~10-min period after aerosolization of beads for 5 min. Data are from 21 mice. r = −0.305, p = 0.001 (MCC vs Time). (**B**) Mucociliary clearance measured over ~10-min period after aerosolization of beads for 15 min, 12 mice were studied. Data from 5 mice in which all beads were plaqued to the tracheal wall, thus exhibiting no MCC, are not plotted. r = −0.0130, p = 0.09. (**C**) Mean MCC rates from mice aerosolized for 5 or 15 min. When non-mobile rafts were observed, they were not included in the mean rate of MCC. However, if no mobile rafts were observed, as was the case for some of the mice receiving aerosol for 15 min (see below), the mean rate of MCC of these mice was included in the average as zero. In all studies, mean data were calculated by determining the average rate of MCC for each mouse and then averaging data for all mice. Mean data in all figures are +/− SEM. **p = 0.001.

Under isoflurane anesthesia, mice were exposed to aerosolized beads for 5 or 15 min, euthanized immediately post-aerosolization, and image acquisition of particle transport was begun. While individual 200 nm beads are too small to visualize using a conventional dissecting scope, *in vivo* the particles quickly coalesced into aggregate “rafts” that were readily detectible with our dissecting scope. The transported beads were in groups of various shapes and sizes from streams to more distinct rafts (Fig. 1A–C). In both the mice receiving aerosol for 5 and 15 min, some of the beads formed various sized plaques on the tracheal wall that often did not move during the measurement period. The rate of MCC was independent of the size of the bead raft. The rate of MCC of individual rafts following 5 min of bead aerosolization in isotonic saline were tracked over a ~10-min period in 21 mice (Fig. 2A). For each mouse about 25 rafts were tracked. A slight but significant decrease in the rate of MCC as a function of time was observed. However, over the first 5 min of measurement, there was no significant effect of time on the rate of MCC.

When we aerosolized beads for 15 min, there was no significant time effect on the rate of raft transport during the 10-min measurement period. In this group of mice, however, we counted significantly fewer groups of mobile rafts/mouse compared to the 5-min aerosol (13.5 vs 25 p = 0.01) in the 10-min measurement period (Fig. 2B), despite a longer aerosolization time which should produce a 3-fold increase in the number of beads deposited. The rafts did not differ noticeably in size or shape between the 5- and 15-min aerosolization times. We hypothesize that the difference in raft counts between 5 and 15-min aerosolization time was due to an increased number of beads plaqued (adhered) to the tracheal walls as a result of the 15-min aerosol. Of note, we observed no measurable transport in 5/12 mice in the 15-min aerosol group, i.e., all visible beads/rafts were plaqued to the tracheal walls, resulting in mean transport rate of 0 in these mice. Since both preparations (5- and 15-min aerosol) appeared to be in a steady state during the first 5 min of MCC measurement, the mean rate of transport was calculated over this time interval. Interestingly, when the beads were aerosolized for 5 min, the rate of MCC was significantly greater than when the beads were aerosolized for 15 min (Fig. 2C).

### Effect of length of anesthesia on MCC

**Figure 3:**
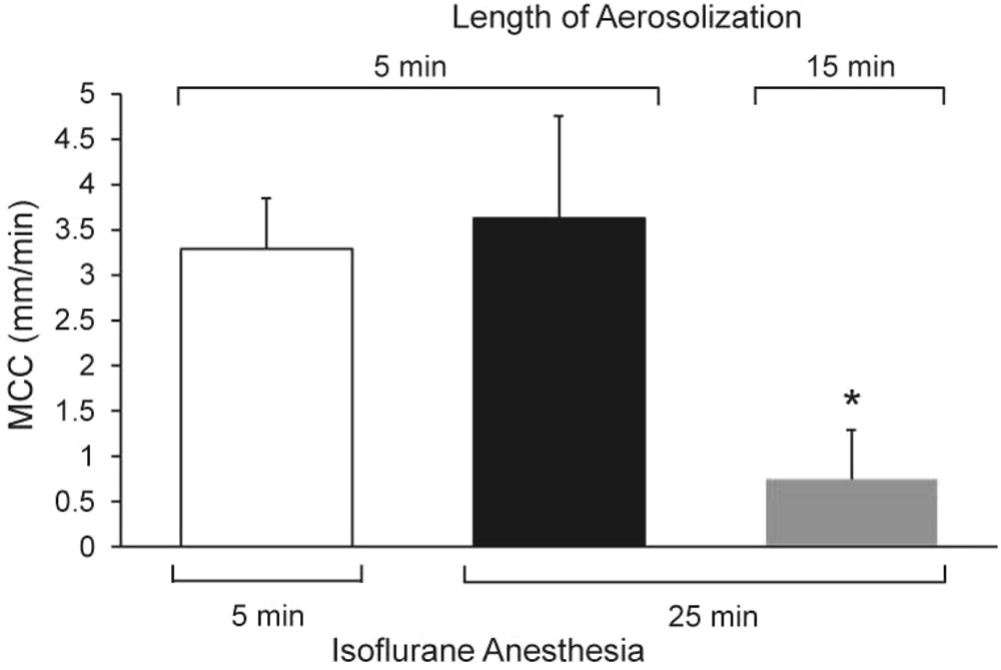
Effect of length of anesthesia on MCC measured in mice anesthetized with isoflurane for either 5 or 25 min and aerosolized with beads for 5 or 15 min. n = 5 mice/group.

Because the mice that were aerosolized for 15 min were also anesthetized for a longer period, we investigated whether the length of isoflurane anesthesia was responsible for the lower rate of MCC in this group of mice. For these experiments, the mice were anesthetized with isoflurane for 5 min, during which time they were also aerosolized with 1x beads, or they were anesthetized with isoflurane for 25 min and aerosolized with 1x beads during the last 5 min of the anesthetic period. These experiments revealed that the length of the anesthetic period had no significant effect on the rate of MCC for beads aerosolized at a 1x concentration (Fig. 3). However, if the mice were anesthetized for 25 min and received the 15-min aerosol, then the rate of MCC was significantly reduced by about 5 fold, p = 0.05 vs 5-min aerosol (25-min isoflurane) (Fig. 3).

### Effect of type anesthetic on MCC

**Figure 4:**
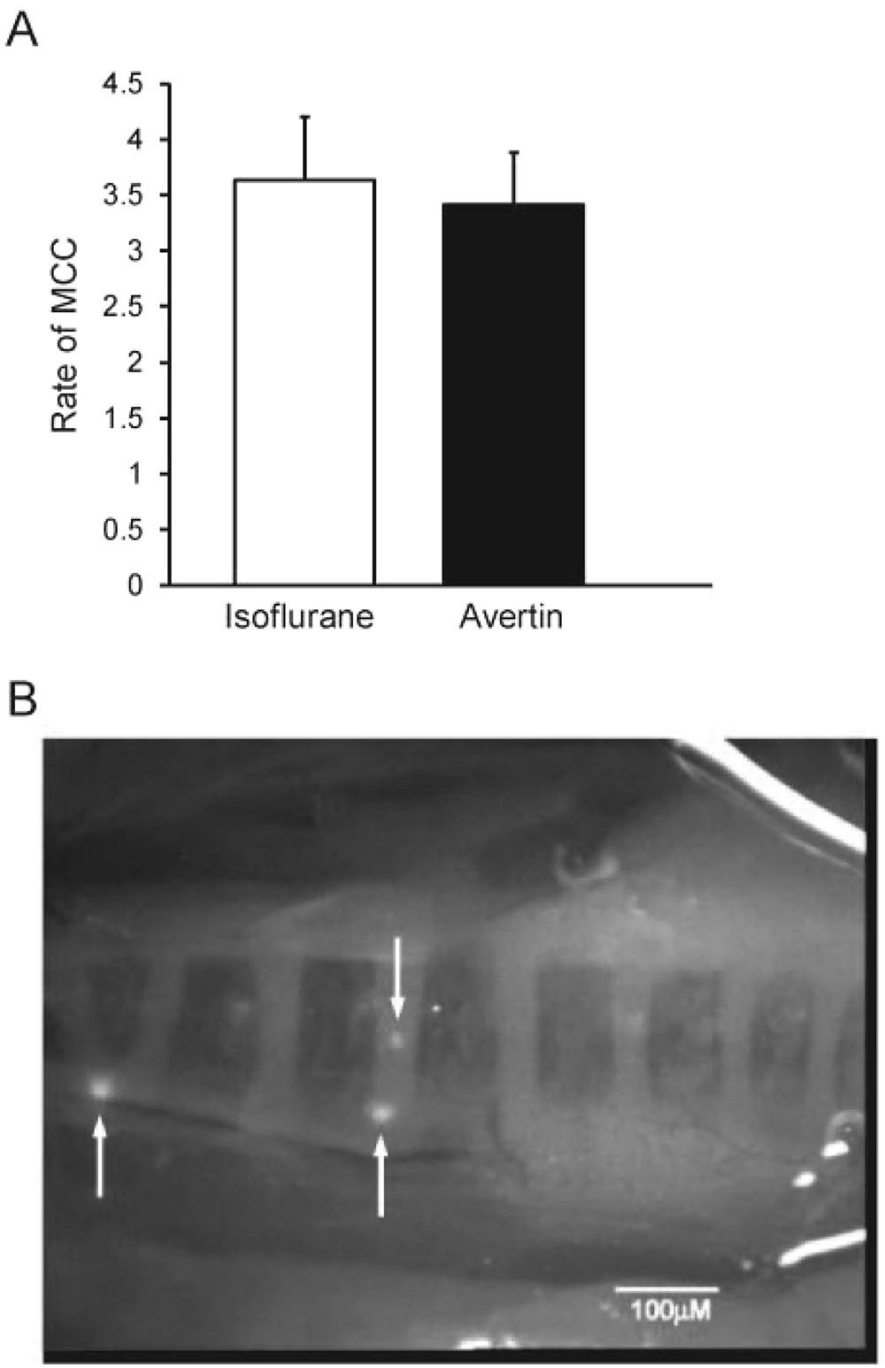
(**A**) Effect of type of anesthetic on MCC. n = 6 isoflurane, n = 9 Avertin. Means +/− SEM. (**B**) Still image captured from video of an Avertin-anesthetized mouse. Arrows indicate mobile rafts, which were significantly smaller than those of the isoflurane-anesthetized mice (compare to Fig. 1). There were almost no immotile beads visible in the Avertin anesthetized mice. The bright areas on the right 1/3 of the image are reflection artifacts.

We investigated the effect of an injectable anesthetic (Avertin) compared to the inhalation anesthetic (isoflurane) on the rate of MCC in a group of C57BL/6 N mice. One group of mice was anesthetized with isoflurane, and once an anesthetic plane of anesthesia was achieved, they were exposed to aerosol with beads (1x isotonic vehicle) for 5 min. A second group was injected with Avertin, and once an anesthetic plane was achieved, they were treated identically. The results, shown in Fig. 4A, indicated that the rate of MCC did not differ between the two anesthetics. Interestingly, however, despite similar transport rates in both groups, we noticed a significantly higher incidence of plaques of beads visible on the tracheal wall of the mice anesthetized with isoflurane (83%, 5/6 of the mice exhibited plaques) vs the mice anesthetized with Avertin (22%, 2/9 formed visible plaques) (p = 0.02). In addition, the transported rafts were significantly smaller in the Avertin-anesthetized mice (Fig. 4B, compare to Fig. 1). (The average surface area of the transported rafts, measured with Image J software (arbitrary units), was 3.99 +/− 0.980 (N = 11) for the isoflurane-anesthetized mice, compared to 0.456 +/− 0.263 (N = 14) for the Avertin-anesthetized mice.) However, the number of rafts detected and counted over the measurement period did not differ significantly between groups (Avertin 16.7 +/− 3.8 vs isoflurane 20.7 +/− 5.7, p = 0.56). The respiratory rates were also markedly different between the two groups. The isoflurane-anesthetized mice had a respiratory rate of ~60 breaths/min, whereas the Avertin-anesthetized mice breathed at a rate of ~130 breaths/min. The tidal volume of the isoflurane mice was enhanced as the mice breathed much more deeply than did the Avertin mice (personal observations). However, despite the low rate of respiration, we have previously determined that the isoflurane mice had normal blood oxygen saturation (unpublished).

### Effect of bead mass delivered

**Figure 5:**
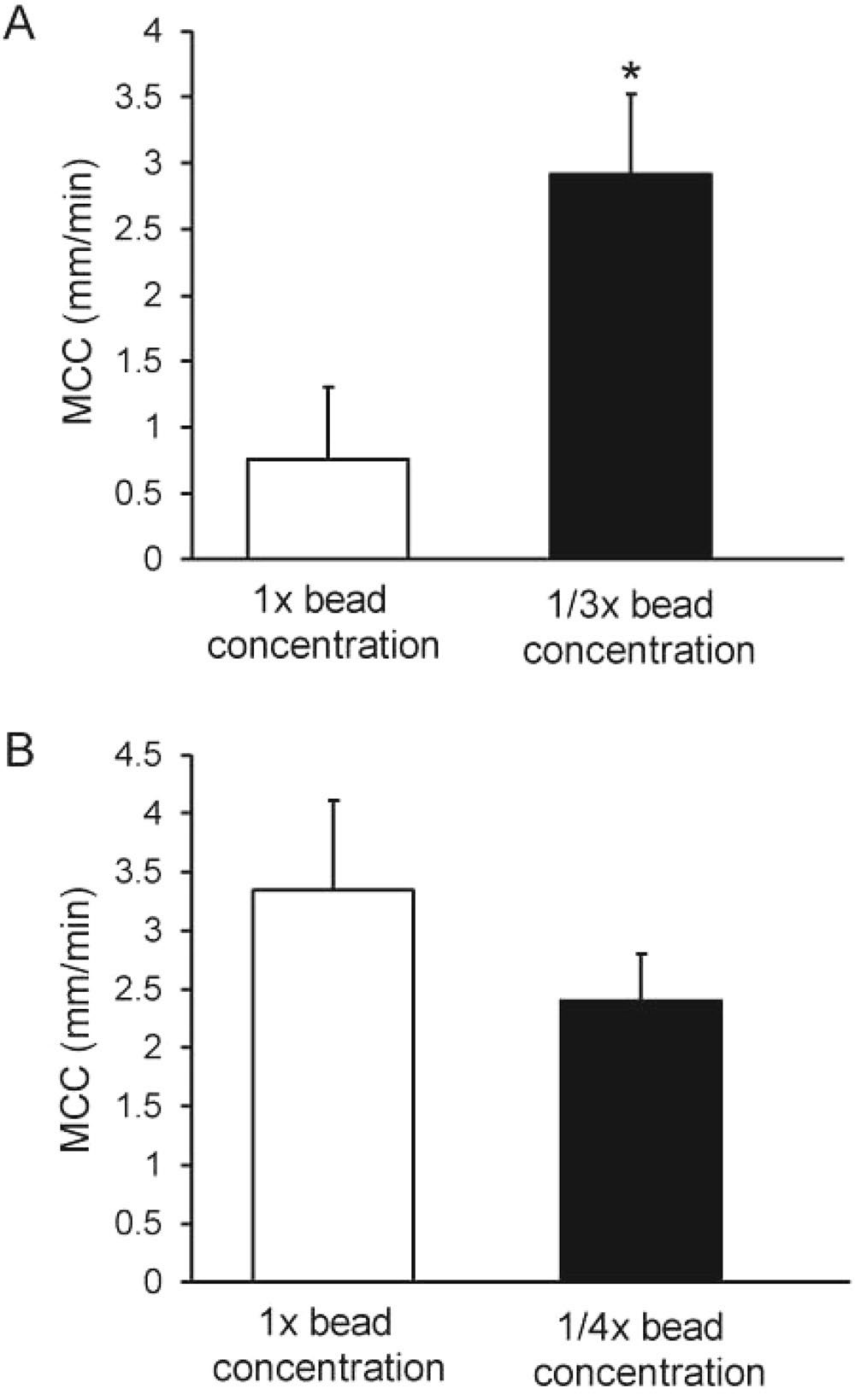
(**A**) Effect of bead mass (15-min aerosol) on MCC. n = 4 mice/group, *p = 0.05. (**B**) Effect of bead mass (5-min aerosol) on the rate of MCC.

While the concentration of the beads delivered to the 5- and 15-min protocols was the same (Fig. 2), the bead mass deposited varied as a function of length of delivery period, i.e., the mice aerosolized for 15 min would have received ~3x the mass of beads delivered to the airways compared to the mice aerosolized for 5 min. To determine the effect of delivered bead mass on MCC independent of length of aerosol, we compared the MCC of beads aerosolized at the usual 1x concentration to that of beads aerosolized at 1/3x concentration, both for 15 min (total 25 min isoflurane anesthesia). We found that the rate of MCC of the 1/3x bead aerosol was significantly elevated compared to the 1x aerosol (Fig. 5A). We next tested whether decreasing the mass of beads administered during 5-min aerosol would affect MCC, by repeating these experiments, decreasing the bead concentration by 75%. We found no significant effect on the rate of MCC in the 5-min aerosolized mice receiving 1x beads compared to mice receiving 1/4x concentration of beads (Fig. 5B). Of note, bead concentration had no effect on aerosol generator output (µl output over time; data not shown).

### Hypertonic NaCl aerosolization

**Figure 6:**
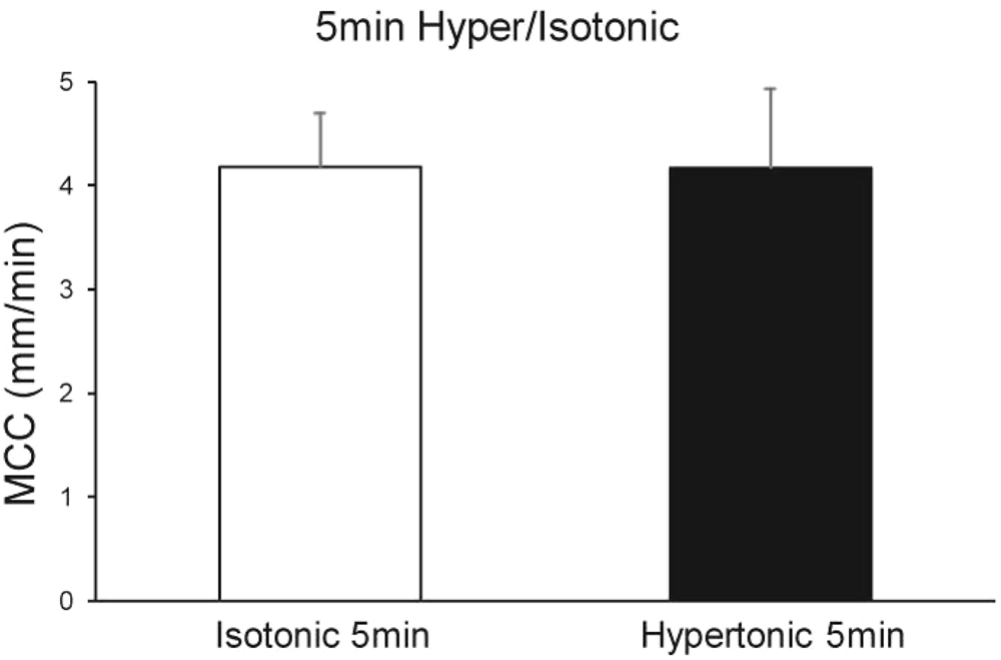
Effect of hypertonic saline (7%) 5-min aerosol on the rate of MCC. N = 5 isotonic mice and n = 6 hypertonic mice.

Because inhaled hypertonic saline has been used clinically to increase the volume of airway surface liquid and restore MCC in patients with CF^18,19^, we determined the effect of hypertonic NaCl on MCC in our murine preparation. We exposed a group of mice for 5 min to an aerosol containing hypertonic saline, 7% NaCl (HS), containing 1x beads and compared the rates of MCC to a group of mice exposed for 5 min to an isotonic NaCl aerosol, 1x beads. As found for the cohort of mice used in the studies shown in Fig. 2A, the smaller group of mice (n = 5) exposed to 1x beads in isotonic NaCl as controls for the HS group exhibited a significant correlation of MCC with time. However, the first 5 min of measurement showed no effect of time. Interestingly, in the HS group, there was no significant correlation of MCC with time over the 10-min measurement period. If we compare the mean rates of MCC in the two groups during the first 5 min, when the rates of MCC were stable in both groups, there was no significant difference in the rate of MCC between the groups (Fig. 6). Interestingly, we counted significantly more rafts/mouse in the isotonic group (21 +/− 2.5 rafts/ mouse) than in the hypertonic group (7.8 +/− 0.95 rafts/mouse, p = 0.026).

### MCC in alive vs dead mice

**Figure 7:**
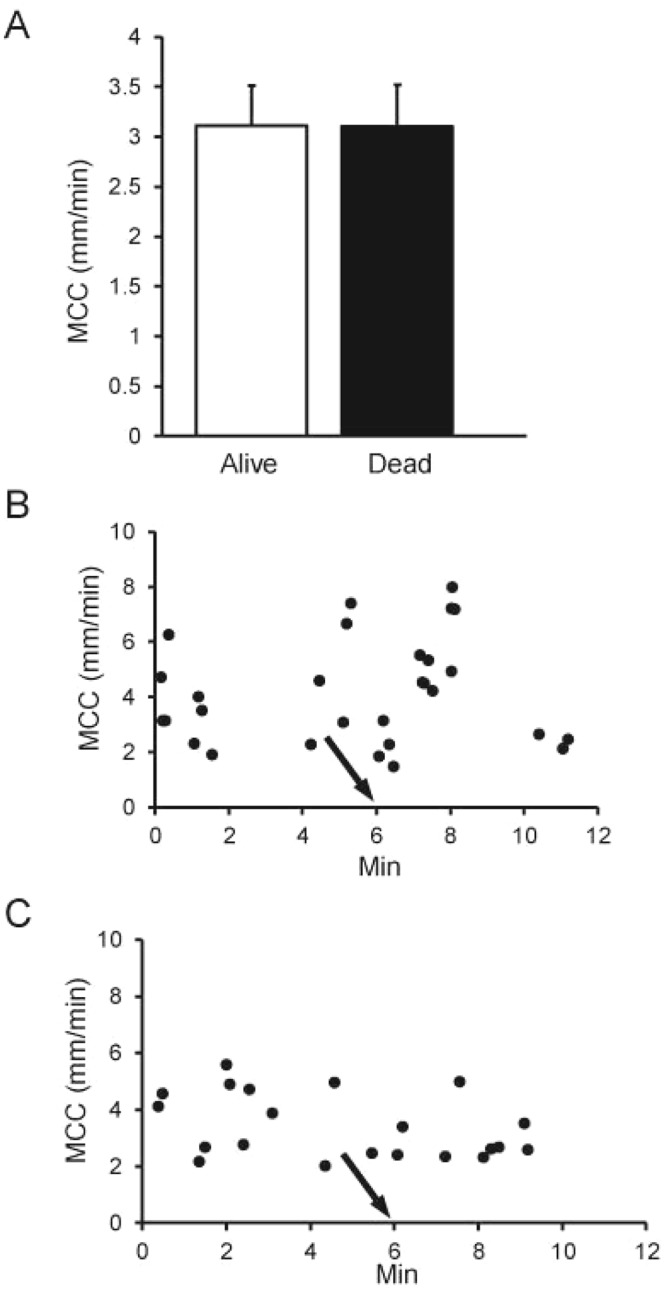
(**A**) MCC in alive or dead mice aerosolized with beads for 5 min (pre-euthanasia). Data are means of 9 mice in each group. (**B**,**C**) MCC data plot of an individual mouse pre and post-euthanasia (arrow). Data points represent individual rafts of beads counted at the times indicated.

For these studies, beads were aerosolized to mice for 5 min under isoflurane anesthesia and MCC of the anesthetized mice was recorded for 5–6 min following aerosolization. Then the mice were euthanized by severing the abdominal aorta, and recording of MCC continued for another 5–6 min. This study revealed that the rate of MCC in the live mice was virtually identical to that following euthanasia (Fig. 7A) (means calculated over the respective measurement period). Two representative data plots are shown in Fig. 7B,C, demonstrating that there was not a significant effect on MCC even immediately after euthanasia. (We have measured MCC for as long as 15 min post-euthanasia without detecting a difference in the rate of MCC between living and dead animals.).

### Effect of UTP

**Figure 8:**
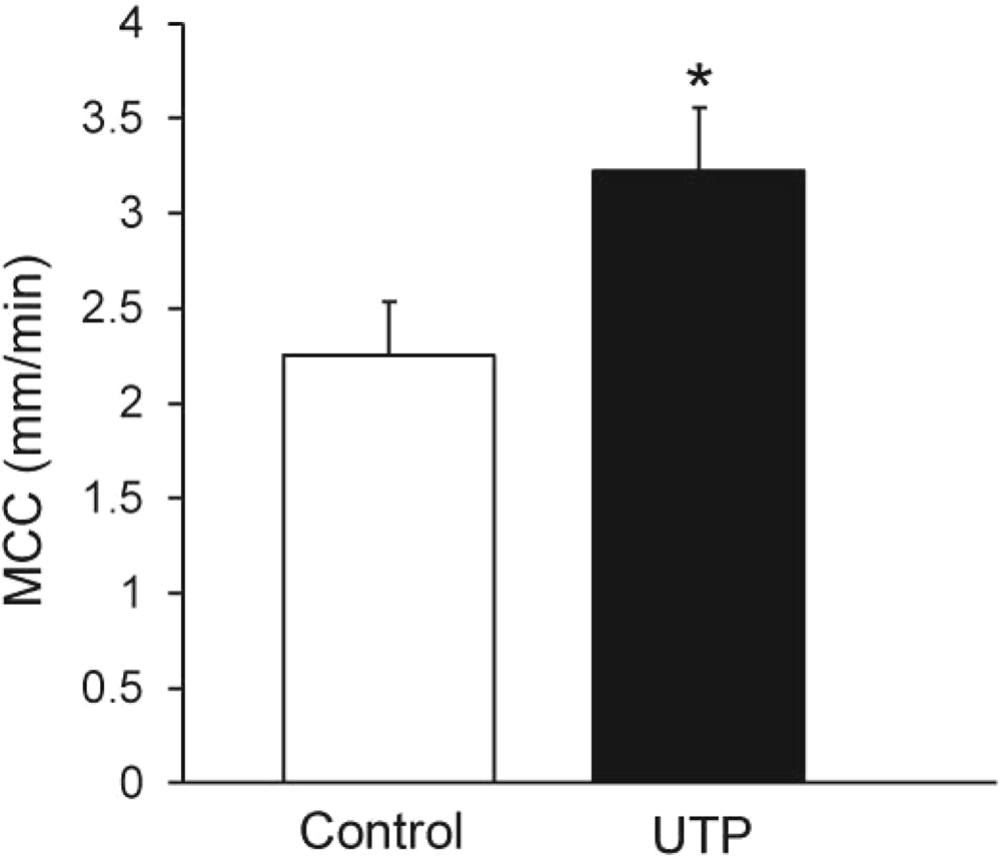
MCC in control mice (non-treated, beads only) and mice given UTP in the 5-min aerosol along with beads. n = 10 mice/group *p = 0.05.

To determine whether incorporation of UTP, a drug known to increase MCC^12,20^, in the aerosol/bead solution would result in an increase in MCC, the mice were exposed to an aerosol for 5 min with 1x beads in isotonic saline +/− UTP at a concentration of 2.5 mM. According to bead deposition and recovery studies previously performed (unpublished), the dilution factor (or the bead concentration on the airway) should be about 0.015% of that in the aerosol generator; thus we would expect a concentration of ~3.75 × 10^−5^M UTP on the airway surface after aerosol administration. UTP administration significantly increased the rate of MCC in these preparations (Fig. 8). It will be noted that the MCC rates for the control mice (1X beads in isotonic NaCl, 5-min aerosol) are less than in preceding figures. The UTP studies and controls were done months after the first studies, so variability in the absolute values is not unexpected.

### Pegylated bead transport

To determine if the beads were sticking to the mucus as a result of an electrical charge, we compared carboxylated nanoparticles with poly(ethylene glycol-PEG) coated nanoparticles (100 nm), which is reported to reduce their affinity for mucus constituents^17^. The rate of transport of these pegylated beads (1x, aerosolized for 5 min) did not differ significantly (3.02 +/− 0.46, n = 5) from identical non-pegylated beads (3.5 +/− 0.37, n = 5). Furthermore, visually the transport of the two beads with respect to plaque formation on the wall of the trachea was indistinguishable.

### MCC in Neonates

We studied a group of neonatal pups using a technique identical to that used for the adult mice. We aerosolized beads (1x, PBS) for 5 min to isoflurane-anesthetized neonatal pups (10 days of age, mean body mass 7.2 +/− 0.31 g). All pups tolerated the isoflurane anesthesia and aerosolization well. The mean rate of MCC was 1.8 +/− 0.064 mm/min (n = 10) in this group of pups. As in the adult mice, we often saw groups of immotile beads plaqued to the tracheal walls in the neonates.

## Discussion

With the advent of genetically engineered mice, it is now possible to study each of the three components of the MCC system (ciliary beat, mucus production, and airways surface hydration) in isolation in order to determine how they interact to effect normal MCC. However, critical to our ability to understand the interrelationship between the components of the MCC system is the ability to accurately measure the rate of MCC in mice.

While a number of studies have measured MCC in mice by a variety of techniques, many of the studies have technical shortcomings and a wide range of MCC rates are reported. Rates of tracheal MCC (or particle transport) for the “normal mouse trachea” reported in the literature differ widely from 15 mm/min^6^ to 0.031 mm/min^21^. Much of the variability reported is likely due to differences in techniques used to measure MCC, in addition to other factors (e.g., strain of mice, age, etc.)^8,14^.

One of the earliest studies reported measured MCC in the intact tracheas of living mice by introducing ~3 μm fluorescent beads into the nasal cavity (15 µl volume) and then measuring transport of the aspirated beads by trans-tracheal fluorescence of the particles^8^. This study found that many mice exhibited no MCC and many beads exhibited a “stop/go” type of transport. This study also found the strain of mice studied significantly influenced the rate of MCC. Others have measured lower airways clearance in the living mouse by scintigraphy after instilling a large volume (~50 μl) of a gamma emitter into the airways^9^. Both of these studies suffer from instilling a relatively large volume of liquid onto the airways and perturbing the volume of airway surface liquid, which has been shown to alter MCC^8,13,22^.

There are a number of studies in the literature in which MCC was measured in the open murine trachea^21,23,24^. While this MCC technique may be technically the easiest, it is likely the most problematic due to the difficulty in maintaining temperature and normal airway hydration, both of which are critically important for normal MCC. MCC rates reported for these preparations range from 0.031 mm/min to 4.5 mm/min^21,24^. MCC in the mouse has also been measured in an excised lung preparation^25^, but this method also used large volumes of liquid, similar to the open tracheal preparations with marked flooding of the excised lung, likely disturbing normal MCC.

An *in vivo* microdialysis technique has been used successfully to identify perturbations in MCC due to genetic manipulation in the lower airways in the mouse^11^. This technique involves placing a very small microdialysis probe on the airway surface^13^ and a small volume of tracer (250 nl) is added distally to the airway epithelium. The time required for the dye to reach the probe is recorded and MCC calculated. While this technique minimizes the amount of exogenous liquid added to the airway surface, it is somewhat time consuming, requires placing a small opening in the trachea, and the placement of the probe may perturb MCC.

Another technique which has been used very successfully to study MCC on a number of genetically engineered mice involves placing a known quantity of fluorescent beads in a very small liquid sample (~150 nl) directly in the lower trachea/mainstem bronchi^14,26–28^. After 15 min, the beads remaining in the trachea/mainstem bronchi are counted and percent clearance is calculated. While only a very small incision is made in the upper trachea to introduce the beads, it cannot be stated with certainty that the airways surface liquid milieu is not disturbed by transient placement of the bead delivery cannula or the small volume of liquid used to introduce the beads. Also, while these studies appear to yield good clearance data and provide clear differences in MCC between WT and mice with mutations expected to perturb MCC^14,26–28^, actual rates of clearance are not obtained with this technique.

A more optimal technique to measure MCC would entail introduction of a tracer in a living mouse without opening the trachea and tracer tracking through the closed trachea. This has been reported by Donnelley *et al*.^16^, but their technique required intubation of the mouse to deliver a dry powder for tracking and expensive equipment for imaging (a synchrotron) which is only available at a few places in the world. Using this set-up, the basal rates of MCC were very low (0.27 mm/min) and the majority of the particles were stationary, likely reflecting airway desiccation as the intubated mice were ventilated with a FlexVent and likely breathing dry air. When the mice were aerosolized for 2.5 min with isotonic NaCl following particle insufflation, the rate of MCC increased significantly and was in the range of 2–3 mm/min, not dissimilar to what we measured in the present investigation (mean MCC with isotonic NaCl in our study was 3.3 mm/min).

A study was recently published in which intubation-free *in vivo* imaging using two-photon microscopy was used to measure MCC in mice^29^. Indeed, the authors found that intubation markedly perturbed accurate MCC measurement and thus concluded that this procedure should be avoided in murine MCC studies. Thus they introduced the tracer into the airways by oropharyngeal aspiration using a relatively large volume of liquid (20 µl). Similar to what we observed in our study, Veres *et al*. report that the fluorescent beads (0.5 um diameter) formed aggregate rafts, which they tracked to determine the over-all rate of MCC (~1.1 mm/min). Although the method used by Veres *et al*. to prepare the trachea for visualization was very similar to the one used in our studies, imaging with two-photon microscopy required a more elaborate and costly set-up compared to our method, which only requires a dissecting scope interfaced with a CCD camera and fluorescent illumination.

Our method to measure MCC delivers the tracer via nasal aerosol inhalation, adding very little volume of liquid to airways (0.095 µl/5-min aerosol), and the trachea remains closed and intact for the MCC measurements. Importantly, we found that the rate of MCC was nearly identical in live mice or shortly after euthanasia. This was rather surprising as we speculated euthanasia might cause adrenergic stimulation (or release of other neurotransmitters) that might stimulate MCC^30^. The absence of respiratory excursion in euthanized mice facilitates particle imaging, and thus MCC measurements on a non-living mouse may be technically easier than performing such measurements on a breathing animal.

We found no significant effect of the length of the isoflurane anesthesia (5 or 25 min) on the rate of MCC. Most of our studies were conducted with mice anesthetized with isoflurane anesthesia, but as access to a dedicated vaporizer might not always be available and simultaneous delivery of isoflurane and an aerosol can be challenging, we conducted a set of studies using the injectable anesthetic, Avertin, which is widely available and not FDA-regulated, as are many injectable anesthetics. The rate of MCC was not different between the two anesthetics. Although the number of bead rafts we counted in the Averetin-anesthetized mice was slightly less than in the isoflurane group, this difference was not significant. Of note, significantly more beads adhered to the tracheal walls in the isoflurane-anesthetized group and the bead rafts in the Avertin mice were significantly smaller than those in the isoflurane mice. We hypothesize that these differences might be due to differences in the quantity of beads deposited in the lungs of the two groups of mice, or in their deposition pattern, especially as the respiratory rate and tidal volumes differed between the mice receiving the two anesthetics.

The length of aerosolization had a significant effect on the rate of MCC, and we determined that this was due to the mass of beads delivered, with shorter aerosolization periods (5 min) giving greater rates of MCC than longer aerosolizations (15 min). It is unlikely that a difference in liquid delivery explains the results because mice aerosolized with 1/3x beads for 15 min received ~3 times as much liquid as did the mice receiving 1x beads for 5 min, and the rates of MCC of these two treatments did not differ. Of note, further dilution of the aerosol beads (1/4x for 5 min, Fig. 5B) did not result in a further increase in MCC. Thus, if too many beads are aerosolized, they appear to overwhelm the transport process and many do not clear. However, even when an optimal mass of beads was aerosolized onto the airway, some of the beads adhered to the walls in many of the preparations. Numerous other investigators have reported beads/particles failure to clear in the murine trachea^8,16,29^.

In contrast, while the size and shapes of the bead rafts were variable within a mouse and between mice, we did not detect a difference in the rate of transport as a function of raft size. Others have also noted that MCC was independent of particle size and shape but was influenced by the material property of the transported particles^31,32^.

Hypertonic saline has been reported to increase the volume of airway surface liquid by osmotically drawing liquid into the airways and has been used clinically to restore/accelerate MCC^18,19^. While we did not see a significant effect of the high salt on the rate of MCC, we did note that the rate of MCC was maintained for the 10-min measurement period in the high salt group, whereas the rate of MCC declined after 5 min in the control group. These data are similar to those reported by Donnelley *et al*.^16^, who observed that although their high salt group did not exhibit a greater rate of MCC as compared to controls treated with isotonic saline, the rate of MCC was maintained for longer periods in the high salt group, suggesting a temporary increase in airway surface hydration. Of note, the studies of Donnelley *et al*. were performed in the presence of a Na^+^ channel blocker added to the high salt group. As our studies were performed without addition of a Na^+^ channel blocker, this supports the hypothesis that hypertonic saline per se can restore the slight decline in MCC observed after 5-min aerosolization with isotonic saline. It is not clear why fewer bead rafts were counted in the high salt group, as there did not appear to be more stationary particles nor a difference in size of the rafts compared to the 5-min isotonic group.

As nucleotides have been demonstrated to increase MCC by increasing CBF, airway surface liquid, and mucus secretion^33^, we investigated the effect of UTP when included in the isotonic 5-min aerosol along with the tracker beads. We found that a 5-min aerosol of UTP significantly increased the rate of bead clearance, suggesting that a dose of UTP sufficient to modify MCC was aerosolized to the airway surfaces. Based on previous studies, it is likely that all three components of the mucociliary clearance system (ciliary beat frequency, mucin secretion, and airway surface hydration), were stimulated by UTP to result in an overall increase in MCC. Thus, our technique will be especially useful for testing various pharmacological preparations that may affect MCC.

Finally, our aerosolization method has been used to successfully measure MCC in 10-day-old neonatal mouse pups. Thus this method will be useful to study MCC during development or in disease models in which some of the mutations that perturb MCC have been found to result in neonatal lethality^11,28^.

In summary, we have developed a simple, inexpensive method to determine MCC in mice *in vivo* (see Supplementary Table S1 for optimal parameters for MCC measurements as determined in this study). Our method uses aerosolization and trans-tracheal tracking of fluorescent beads for MCC determination and has a number of advantages over other MCC methods reported in the literature. First, the trachea does not have to be opened (or intubated) for introduction of the tracer, and only a very small volume of liquid is inhaled with the tracer beads, reducing the likelihood of perturbing the milieu of the airway surface liquid. Second, we can actually visualize the behavior of the beads during clearance, which can provide additional information about the changes introduced by a particular intervention, i.e., migration patterns, clumping, and/or deviation from uniform trajectories, in addition to an actual rate of MCC. Third, we have successfully used this method to study MCC in very young mouse pups. Moreover, since this method is not necessarily terminal, it can be performed on living mice and it can be used to study MCC pre- and post-drug treatment. In addition, we obtain nearly identical rates of MCC on living or post-euthanasia mice. Finally, the equipment required for these studies is relatively inexpensive and easily obtainable and hopefully will allow labs that study perturbations in the components of the MCC system to actually measure MCC in these mice.

## Electronic supplementary material

Supplementary Video S1

Supplementary Video S2

Supplementary Video S3

Supplementary Information

## Data Availability

The datasets generated and/or analyzed during the current study are available from the corresponding author on reasonable request.

## References

[1] Bustamante-Marin Ximena M., Ostrowski Lawrence E. (2016). Cilia and Mucociliary Clearance. Cold Spring Harbor Perspectives in Biology.

[2] Knowles MR, Boucher RC (2002). Mucus clearance as a primary innate defense mechanism for mammalian airways. J Clin Invest.

[3] Azar A, Piccinelli C, Brown H, Headon D, Cheeseman M (2016). Ectodysplasin signalling deficiency in mouse models of hypohidrotic ectodermal dysplasia leads to middle ear and nasal pathology. Hum Mol Genet.

[4] Gilley SK (2014). Deletion of airway cilia results in noninflammatory bronchiectasis and hyperreactive airways. Am J Physiol Lung Cell Mol Physiol.

[5] Liu Y (2013). Increased susceptibility to pulmonary Pseudomonas infection in Splunc1 knockout mice. J Immunol.

[6] Hussong J (2013). Cilia-driven particle and fluid transport over mucus-free mice tracheae. J Biomech.

[7] Scott DW (2017). SPX-101 Is a Novel Epithelial Sodium Channel-targeted Therapeutic for Cystic Fibrosis That Restores Mucus Transport. Am J Respir Crit Care Med.

[8] Brownstein DG (1987). Tracheal mucociliary transport in laboratory mice: evidence for genetic polymorphism. Exp Lung Res.

[9] Foster WM, Walters DM, Longphre M, Macri K, Miller LM (2001). Methodology for the measurement of mucociliary function in the mouse by scintigraphy. J Appl Physiol.

[10] Kerem E (1999). Pulmonary epithelial sodium-channel dysfunction and excess airway liquid in pseudohypoaldosteronism. N Engl J Med.

[11] Mall M, Grubb BR, Harkema JR, O’Neal WK, Boucher RC (2004). Increased airway epithelial Na+ absorption produces cystic fibrosis-like lung disease in mice. Nat Med.

[12] Begrow F, Verspohl EJ (2013). Effect of Ap4A, UTP and salbutamol on mucociliary clearance in a mouse model of cystic fibrosis (*in situ*). Pharmacol Pharm.

[13] Grubb BR, Jones JH, Boucher RC (2004). Mucociliary transport determined by *in vivo* microdialysis in the airways of normal and CF mice. Am J Physiol Lung Cell Mol Physiol.

[14] Grubb BR, Livraghi-Butrico A, Rogers TD, Yin W, Ostrowski LE (2016). Reduced mucociliary clearance in old mice is associated with a decrease in Muc5B mucin. Am J Physiol Lung Cell Mol Physiol.

[15] Livraghi-Butrico A (2017). Contribution of mucus concentration and secreted mucins Muc5ac and Muc5b to the pathogenesis of muco-obstructive lung disease. Mucosal Immunol.

[16] Donnelley M (2014). Non-invasive airway health assessment: synchrotron imaging reveals effects of rehydrating treatments on mucociliary transit *in-vivo*. Sci Rep.

[17] Mert O (2012). A poly(ethylene glycol)-based surfactant for formulation of drug-loaded mucus penetrating particles. J Control Release.

[18] Donaldson SH (2006). Mucus clearance and lung function in cystic fibrosis with hypertonic saline. N Engl J Med.

[19] Elkins MR (2006). A controlled trial of long-term inhaled hypertonic saline in patients with cystic fibrosis. N Engl J Med.

[20] Olivier KN (1996). Acute safety and effects on mucociliary clearance of aerosolized uridine 5′-triphosphate +/− amiloride in normal human adults. Am J Respir Crit Care Med.

[21] Zahm JM (1997). Early alterations in airway mucociliary clearance and inflammation of the lamina propria in CF mice. Am J Physiol.

[22] Gatto LA (1993). Cholinergic and adrenergic stimulation of mucociliary transport in the rat trachea. Respir Physiol.

[23] Konig P, Krain B, Krasteva G, Kummer W (2009). Serotonin increases cilia-driven particle transport via an acetylcholine-independent pathway in the mouse trachea. PLoS One.

[24] Look DC (2001). Effects of paramyxoviral infection on airway epithelial cell Foxj1 expression, ciliogenesis, and mucociliary function. Am J Pathol.

[25] Cowley EA, Wang CG, Gosselin D, Radzioch D, Eidelman DH (1997). Mucociliary clearance in cystic fibrosis knockout mice infected with Pseudomonas aeruginosa. Eur Respir J.

[26] Chen Gang, Volmer Allison S., Wilkinson Kristen J., Deng Yangmei, Jones Lisa C., Yu Dongfang, Bustamante-Marin Ximena M., Burns Kimberlie A., Grubb Barbara R., O’Neal Wanda K., Livraghi-Butrico Alessandra, Boucher Richard C. (2018). Role of Spdef in the Regulation of Muc5b Expression in the Airways of Naive and Mucoobstructed Mice. American Journal of Respiratory Cell and Molecular Biology.

[27] Donoghue LJ (2017). Identification of trans protein QTL for secreted airway mucins in mice and a causal role for Bpifb1. Genetics.

[28] Ostrowski LE (2010). Conditional deletion of dnaic1 in a murine model of primary ciliary dyskinesia causes chronic rhinosinusitis. Am J Respir Cell Mol Biol.

[29] Veres TZ (2017). Intubation-free *in vivo* imaging of the tracheal mucosa using two-photon microscopy. Sci Rep.

[30] Camner P, Strandberg K, Philipson K (1976). Increased mucociliary transport by adrenergic stimulation. Arch Environ Health.

[31] Henning A (2010). Influence of particle size and material properties on mucociliary clearance from the airways. J Aerosol Med Pulm Drug Deliv.

[32] Kirch J (2012). Mucociliary clearance of micro- and nanoparticles is independent of size, shape and charge–an *ex vivo* and *in silico* approach. J Control Release.

[33] Davis CW, Lazarowski E (2008). Coupling of airway ciliary activity and mucin secretion to mechanical stresses by purinergic signaling. Respir Physiol Neurobiol.

