# Supplementary material for: Mucociliary Clearance in Mice Measured by Tracking Trans-tracheal Fluorescence of Nasally Aerosolized Beads
Source: Sci Rep. 2018 Oct 3;8:14744. doi: 10.1038/s41598-018-33053-2 (PMC6170422; doi:10.1038/s41598-018-33053-2)
Supplement: Supplementary file 4 — Supplementary Information [file 41598_2018_33053_MOESM4_ESM.docx]

**Mucociliary Clearance in Mice Measured by Tracking Trans-tracheal Fluorescence of Nasally Aerosolized Beads**

Troy D. Rogers, Lawrence E. Ostrowski, Alessandra Livraghi-Butrico, Brian Button, and Barbara R Grubb

**Supplementary Information**

**Supplementary Table S1.** Optimal parameters for MCC measurement as determined by the present investigation

| Anesthesia | Avertin or isoflurane |
| --- | --- |
| Aerosol generator | Adapted Aeroneb Pro  (or commercially available aerosol generators suitable for mice; e.g., Data Scientific International St. Paul, MN.; Harvard Apparatus, Holliston MA.; Braintree Scientific Inc., Braintree MA.) Output of our generator was 9.7 +/- 0.44 ul/min. |
| Aerosol delivery nozzle | (I.D 4 mm) positioned 5 mm above anesthetized mouse’s nose |
| Beads | 200 nm fluorescent, optimal concentration 2% solids (ThermoFisher, In Vitrogen) |
| Optimal aerosol time | 5 min |
| Optimal video bead tracking | 5-10 min |

**Supplementary Video S1.** Mouse was aerosolized with isotonic saline for 15 min, 1x beads. Some mobile rafts and stationary beads are visible

**Supplementary Video S2.** Mouse was aerosolized with isotonic saline for 5 min, 1x beads.

**Supplementary Video S3.** Mouse was aerosolized with isotonic saline for 5 min 1X beads. Most beads are mobile but some small non-mobile plaques are visible.

**
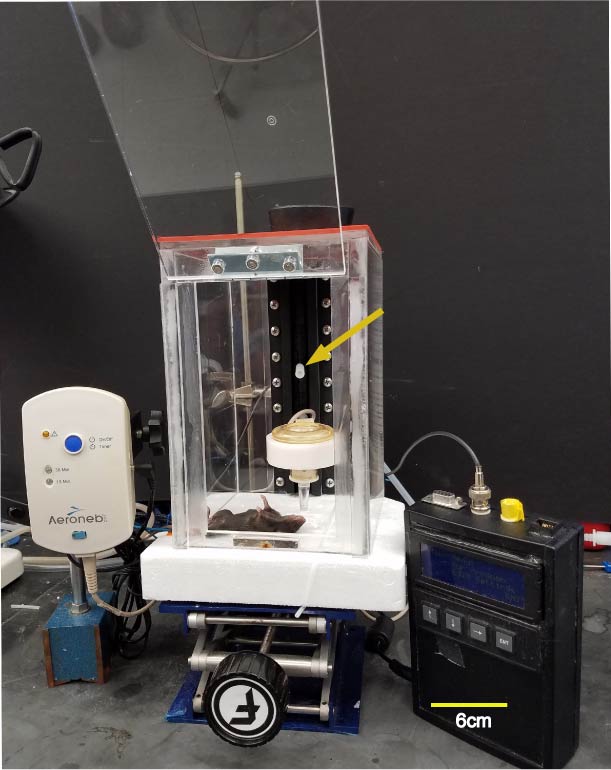
**

**Supplementary Figure S1.** Actual set-up used to deliver aerosol to the mouse. The yellow line indicates the isoflurane delivery tube in the chamber. The door to the chamber is in the open position, but is closed during aerosolization. The rectal temperature probe and mouse’s tail is positioned in a grove on the Styrofoam base under the plexiglass box.


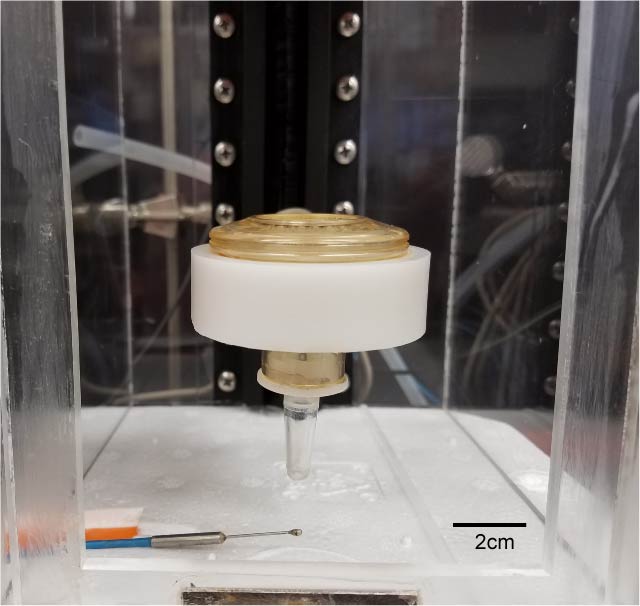


**Supplementary Figure S2.** Close up of the aerosol generator and the nozzle tip (I.D 4mm).
